# Mismatch Amplification Mutation Assay-Based Real-Time PCR for Rapid Detection of Neisseria gonorrhoeae and Antimicrobial Resistance Determinants in Clinical Specimens

**DOI:** 10.1128/JCM.00365-18

**Published:** 2018-08-27

**Authors:** Valentina Donà, Joost H. Smid, Sara Kasraian, Dianne Egli-Gany, Ferah Dost, Fatime Imeri, Magnus Unemo, Nicola Low, Andrea Endimiani

**Affiliations:** a Institute for Infectious Diseases, University of Bern, Bern, Switzerland; b Institute of Social and Preventive Medicine, University of Bern, Bern, Switzerland; c Ambulatorium Kanonengasse, Zurich, Switzerland; d Laborgemeinschaft 1, Zurich, Switzerland; e WHO Collaborating Centre for Gonorrhoea and Other STIs, Örebro University, Örebro, Sweden; Marquette University

**Keywords:** gonococcus, antimicrobial resistance, NAAT, real-time PCR, clinical samples, AMR, Neisseria gonorrhoeae, antibiotic resistance, clinical methods, diagnostics, rapid tests, sexually transmitted diseases

## Abstract

Molecular methods are often used for Neisseria gonorrhoeae detection, but complete definition of antimicrobial resistance (AMR) patterns still requires phenotypic tests. We developed an assay that both identifies N. gonorrhoeae and detects AMR determinants in clinical specimens. We designed a mismatch amplification mutation assay (MAMA)-based SYBR green real-time PCR targeting one N. gonorrhoeae-specific region (*opa*); mosaic *penA* alleles (Asp345 deletion [Asp345del], Gly545Ser) associated with decreased susceptibility to cephalosporins; and alterations conferring resistance to ciprofloxacin (GyrA Ser91Phe), azithromycin (23S rRNA A2059G and C2611T), and spectinomycin (16S rRNA C1192T). We applied the real-time PCR to 489 clinical specimens, of which 94 had paired culture isolates, and evaluated its performance by comparison with the performance of commercial diagnostic molecular and phenotypic tests. Our assay exhibited a sensitivity/specificity of 93%/100%, 96%/85%, 90%/91%, 100%/100%, and 100%/90% for the detection of N. gonorrhoeae directly from urethral, rectal, pharyngeal, cervical, and vaginal samples, respectively. The MAMA strategy allowed the detection of AMR mutations by comparing cycle threshold values with the results of the reference *opa* reaction. The method accurately predicted the phenotype of resistance to four antibiotic classes, as determined by comparison with the MIC values obtained from 94 paired cultures (sensitivity/specificity for cephalosporins, azithromycin, ciprofloxacin, and spectinomycin resistance, 100%/95%, 100%/100%, 100%/100%, and not applicable [NA]/100%, respectively, in genital specimens and NA/72%, NA/98%, 100%/97%, and NA/96%, respectively, in extragenital specimens). False-positive results, particularly for the *penA* Asp345del reaction, were observed predominantly in pharyngeal specimens. Our real-time PCR assay is a promising rapid method to identify N. gonorrhoeae and predict AMR directly in genital specimens, but further optimization for extragenital specimens is needed.

## Abstract (precis)

Molecular methods are often used for Neisseria gonorrhoeae detection, but complete definition of antimicrobial resistance (AMR) patterns still requires phenotypic tests. We developed an assay that both identifies N. gonorrhoeae and detects AMR determinants in clinical specimens.

## INTRODUCTION

Neisseria gonorrhoeae is the causative agent of the sexually transmitted infection gonorrhea. More than 90% of the estimated 78 million new cases of gonorrhea each year occur in low- and middle-income countries, where access to diagnosis and treatment is often limited (1). Despite international efforts to promote preventive strategies and ensure adequate treatment, N. gonorrhoeae may develop mechanisms of resistance to all antimicrobials that have been recommended for empirical first-line treatment (2–4).

Commercial nucleic acid amplification tests (NAATs) have replaced culture-based methods for the diagnosis of gonococcal infections in many settings. However, a recognized disadvantage of these assays is that they do not provide any information about antimicrobial resistance (AMR) patterns (5). Culture methods are time-consuming and limited by stringent sampling, transport, and culture conditions, but they remain essential for surveillance to identify changes in AMR trends, detect emerging resistance patterns, and inform prompt revision of treatment guidelines (6, 7). The importance of developing NAATs that detect and predict AMR determinants has been clearly recognized, both to enhance culture-based AMR surveillance and, potentially, to support clinicians to provide individualized therapies (5, 8–10).

Most key molecular mechanisms that confer resistance to currently and previously recommended antimicrobials for gonorrhea are known. The presence of mosaic *penA* alleles encoding penicillin binding protein 2 (PBP2) has been associated with decreased susceptibility and resistance to extended-spectrum cephalosporins (ESCs). For instance, the mosaic *penA* XXXIV allele, present in the successful internationally spreading clone with N. gonorrhoeae multiantigen sequence typing (NG-MAST) sequence type 1407 (ST1407), as well as in other strains belonging to the same genogroup (11–14), has resulted in clinical treatment failures with ESCs worldwide (15). A Ser91Phe substitution in GyrA is present in all documented ciprofloxacin (CIP)-resistant gonococcal strains (16). Moderate- and high-level resistance to azithromycin (AZM) is associated with the single nucleotide polymorphism (SNP) C2611T or A2059G in at least three of the four 23S rRNA alleles (17, 18). The SNP C1192T in the 16S rRNA gene confers spectinomycin (SPC) resistance (19).

Many PCR-based tests to detect molecular determinants predicting resistance to a single antibiotic have been developed, but there have been only a few published attempts to develop assays that predict AMR to multiple drugs (20). We previously developed a multiplex high-resolution melting (HRM)-based real-time PCR to detect N. gonorrhoeae and seven genetic AMR determinants in clinical isolates (21). However, cross-amplification of Neisseria spp. and the relatively high limit of detection (LOD) suggested that our assay would not be suitable for direct testing of clinical specimens.

To overcome the limitations of our previously presented assay (20), we developed a new rapid SYBR green-based real-time PCR method based on a mismatch amplification mutation assay (MAMA) strategy (22). This approach relies on strategic primer design to selectively amplify a target sequence only in the presence of the AMR mutation of interest while suppressing wild-type amplification. The objectives of this study were to describe our new MAMA-based SYBR green real-time PCR and report on its performance for the identification (ID) of N. gonorrhoeae and detection of gonococcal AMR determinants directly in clinical specimens.

## MATERIALS AND METHODS

### Design of the real-time PCR assay.

Eight primer sets were designed with Oligo Primer Analysis software (v4.0; Molecular Biology Insights) to amplify specific sequences of the targets described in Table 1. For ID, primers that amplify the gonococcal upstream regions of *opa* and *porA* were designed (21).

**TABLE 1:** Primers implemented for the real-time PCR assay, target mutations, and the corresponding affected antibiotics^*a*^

Target	Primer name	Primer sequence^*b*^	Associated target (purpose or antibiotic affected)
*opa*	opa_F	5′-GTTCATCCGCCATATTGTGTTGA-3′	*opa* (Neisseria gonorrhoeae identification)
	opa_R	5′-AAGGGCGGATTATATCGGGTTCC-3′	
*porA*	porA_F	5′-CTATGCCCATGGTTTCGACTTTG-3′	*porA* (N. gonorrhoeae identification, confirmatory)
	porA_R	5′-CATAGCTGGTATGTTCGCGTTTC-3′	
*penA* Gly545Ser	545_F	5′-TGGTTAACGGTCGTTACGTCGATT-3′	Mosaic *penA* (decreased susceptibility/resistance to ESCs)
	545_R	5′-GGCCCTGCCACTACACCGTT-3′	
*penA* Asp345del	345_F	5′-GGCAAAGTGGATGCAACCGAT-3′	Mosaic *penA* (decreased susceptibility/resistance to ESCs)
	345_R	5′-GATAAACGTGGGTATCTTGTACGG-3′	
23S rRNA C2611T	C2611_F	5′-AACGTCGTGAGACAGTTTGGTTT-3′	23S rRNA C2611T (moderate AZM resistance)
	C2611_R	5′-GAACTTAGCTACCCGGCTATGCA-3′	
23S rRNA A2059G	A2059_F	5′-TACAGTAAAGGTTCACGGGGTCAC-3′	23S rRNA A2059G (high AZM resistance)
	A2059_R	5′-ATGGCCACACTGTCTCCTCCC-3′	
*gyrA* Ser91Phe	gyrA_S91_F	5′-AATACCACCCCCACGGCGATCT-3′	GyrA Ser91Phe (CIP resistance)
	gyrA_S91_R	5′-TCTATCAGCACATAACGCATAGCG-3′	
16S rRNA C1192T	16S_1192_F	5′-CTTGTCATTAGTTGCCATCATTTG-3′	16S rRNA C1192T (SPC resistance)
	16S_1192_R	5′-TAAGGGCCATGAGGACTTGATA-3′	

a ESCs, extended-spectrum cephalosporins; AZM, azithromycin; CIP, ciprofloxacin; SPC, spectinomycin.

b All nontemplate base pairs compared to the wild-type sequence are underlined.

For the AMR determinants, one primer was designed to match at its 3′ end the mutations of interest in the *penA*, *gyrA*, 23S rRNA, and 16S rRNA genes, and a mismatch in the second to the last base pair at the 3′ end was added. This strategy was also applied to the lower primer of the 16S rRNA reaction to improve discrimination from other Neisseria spp. For both mosaic *penA* reactions (reactions for the *penA* Asp345 deletion [Asp345del] and *penA* Gly545Ser), the primer design also took advantage of mosaic/allele-specific regions in *penA*.

### Neisseria control strains for method validation.

Extraction of genomic DNA (gDNA) from culture strains for validation of the real-time PCR method was performed using a QIAamp DNA minikit (Qiagen). Each 20-μl reaction mixture contained 0.2 μM each primer, 1× MeltDoctor master mix (Applied Biosystems) containing the SYBR green derivative Syto, and 20 ng (or defined copy numbers) of gDNA. Each sample was tested in duplicate. Experiments were run on a QuantStudio 7 Flex instrument (Applied Biosystems). The PCR program parameters included an initial denaturation step (95°C, 10 min), followed by 40 cycles of denaturation (95°C, 15 s), annealing (62°C, 10 s), and extension (72°C, 10 s). Results were analyzed with QuantStudio 6 and 7 Flex real-time PCR software (v1.0; Applied Biosystems). To assess the LOD of the method, known quantities of gDNA copy numbers per reaction mixture were tested in 10-fold serial dilutions.

We tested a panel of 45 N. gonorrhoeae strains that included 26 previously fully characterized strains with known profiles of MICs and genetic AMR determinants (23); GC3 (with the 16S rRNA C1192T mutation; SPC MIC, >1,024 μg/ml); GC4 (harboring four 23S rRNA alleles with the A2059G mutation; AZM MIC, ≥256 μg/ml); G07 (harboring four 23S rRNA alleles with the C2611T mutation; AZM MIC, 8 μg/ml) (21); the fully susceptible reference strain ATCC 49226; and WHO reference strains (24) WHO A; WHO F; WHO G; WHO L; WHO K and WHO W (WHO K and WHO W carry a mosaic *penA* X allele; MIC of cefixime [CFM], 0.25 and 0.5 μg/ml, respectively); WHO M; WHO N; WHO O (with the 16S rRNA C1192T mutation; SPC MIC, >1,024 μg/ml); WHO P; WHO U (harboring four 23S rRNA alleles with the C2611T mutation; AZM MIC, 4 μg/ml); WHO V (harboring four 23S rRNA alleles with the A2059G mutation; AZM MIC, ≥256 μg/ml), WHO Y (carrying a mosaic *penA* XXXIV with an additional Ala501Pro alteration; MICs for CFM and ceftriaxone [CRO], 2 and 1.5 μg/ml, respectively); and WHO X and WHO Z (both of which carry a mosaic *penA* allele; MICs for CFM and CRO, 4 and 2 μg/ml, respectively, for WHO X and 2 and 0.5 μg/ml, respectively, for WHO Z) (24, 25). All N. gonorrhoeae isolates were grown on chocolate agar PolyVitex plates (bioMérieux) for 24 h at 35°C in a humid 5% CO_2_-enriched atmosphere.

Twenty clinical nongonococcal Neisseria strains previously identified by matrix-assisted laser desorption ionization–time of flight mass spectrometry (MALDI-TOF MS; Bruker Daltonics) were also tested to assess cross-reactivity. The panel included N. meningitidis (*n* = 3), N. sicca (*n* = 3), N. flavescens (*n* = 3), N. mucosa (*n* = 2), N. cinerea (*n* = 2), N. subflava (*n* = 2), N. lactamica (*n* = 1), N. flava (*n* = 1), N. weaveri (*n* = 1), N. polysaccharea (*n* = 1), and N. bacilliformis (*n* = 1) strains.

### N. gonorrhoeae-positive and N. gonorrhoeae-negative clinical specimens.

From May 2015 to June 2016, we collected 819 clinical specimens, using ESwabs (Copan Diagnostics Inc.), from 261 patients at three study centers in Switzerland (Checkpoint Zurich, Zurich, Switzerland; Ambulatorium Kanonengasse, Zurich, Switzerland; Department of Infectious Diseases, Bern University Hospital, Bern, Switzerland). Our study population consisted of 240 (92.0%) men, of which 230 (88.1%) were men who have sex with men (MSM), and 21 (8.0%) women. This study was conducted according to the Declaration of Helsinki and was approved by the ethics committees in Bern and Zurich (EK BE 244/14 and EK ZH 2014-0587, respectively).

These samples were tested for N. gonorrhoeae by culture and by the Aptima Combo 2 assay (Hologic Inc.) (*n* = 238 patients) at the laboratory Laborgemeinschaft 1 (Zurich, Switzerland) or by the Cobas CT/N. gonorrhoeae assay (Roche Molecular Diagnostics) (*n* = 23 patients) at the Clinical Microbiology Laboratory of the Institute for Infectious Diseases, University of Bern, Bern, Switzerland (see Table S1 in the supplemental material). After routine testing and storage at −80°C, residual ESwabs and cultures were transported periodically (i.e., approximately every 2 months for samples from the laboratory of Laborgemeinschaft 1) to the Institute for Infectious Diseases (University of Bern, Bern, Switzerland) and stored at −80°C before implementation of the new MAMA-based SYBR green real-time PCR.

A QIAamp DNA minikit (Qiagen) was used to extract total DNA from 200 μl of the residual ESwab specimens which met the minimum requirements for further testing with our method. Reasons for exclusion included the following: <200 μl sample was left, transport/delivery was delayed, the samples could not be tested immediately after DNA extraction, and/or the extragenital samples could not be tested immediately after arrival. For each PCR, 4 μl of extracted total DNA was used, and the real-time PCR was performed as described above.

### Establishment of cycle threshold (*C_T_*) ranges for the detection of AMR determinants.

Detection of mutations in the target genes was based on the difference in the cycle threshold (Δ*C_T_*) values between the reference *opa* reaction and each AMR determinant reaction. We used the N. gonorrhoeae control isolates (tested in duplicate) to determine the optimal Δ*C_T_* for the detection of each AMR determinant using the Youden index, which maximized the sum of sensitivity and specificity (26). We then rounded the value to the nearest whole number and added 1 to account for intersample variability. Adding 1 minimized the number of false-negative results when applied to the clinical samples, which tended to have Δ*C_T_* values higher than the ones observed for culture isolates (data not shown).

### Establishment of the *C_T_* cutoff point for the detection of N. gonorrhoeae in clinical specimens.

We used the results of the commercial NAATs as the reference standard and determined the cutoff point for the *opa C_T_* value to classify positive and negative results. In particular, we examined the distributions of *C_T_* values for specimens with positive and negative reference test results and determined the optimal cutoff point that maximized the sum of the sensitivity and the specificity for the detection of N. gonorrhoeae (26).

### Analysis of the real-time PCR results in clinical specimens.

For N. gonorrhoeae identification, results from the *opa* reaction were interpreted as positive if the *C_T_* value was below the cutoff point. We calculated the sensitivity and the specificity with 95% confidence intervals (CI; determined using the Clopper-Pearson method) by comparison with the results of commercial NAAT testing as the reference standard (27).

For the detection of AMR determinants, the results were interpreted as follows: (i) a positive *penA* Asp345del reaction (indicating the presence of a mosaic *penA* allele) without or with a positive *penA* Gly545Ser reaction (indicating the presence of a *penA* mosaic XXXIV) for strains with decreased susceptibility to CFM and/or CRO; (ii) the presence of the 23S rRNA C2611T or A2059G mutation for strains moderately or highly resistant to AZM, respectively; (iii) the presence of *gyrA* with the Ser91Phe substitution for strains nonsusceptible to CIP; and (iv) the presence of the 16S rRNA C1192T mutation for strains resistant to SPC.

We calculated the sensitivity and the specificity with the 95% CI of the real-time PCR AMR reactions using as the reference standard either their molecular characterization or the antimicrobial susceptibility based on the MICs for CRO, CFM, AZM, CIP, and SPC of culture isolates. MICs were obtained with the Etest (bioMérieux) method on chocolate agar PolyVitex plates (bioMérieux) and categorized using the 2016 European Committee on Antimicrobial Susceptibility Testing (EUCAST) criteria for all antimicrobials except AZM, for which a breakpoint of an MIC of >2 μg/ml was used, as described previously (21, 28–30).

## RESULTS AND DISCUSSION

### Method design and validation using control strains.

To detect SNPs in the 23S rRNA, *gyrA*, and 16S rRNA genes associated with resistance to AZM, CIP, and SPC, respectively (16–19), we used the MAMA method to design primers to selectively amplify mutated alleles while suppressing wild-type amplification (Table 1) (22). Briefly, the sequence of one primer matched at the 3′ end the mutation of interest, and a nontemplate base was introduced in the second to the last base at the 3′ end. Based on previous observations (22), this was expected not to influence the amplification of a mutated allele while strongly inhibiting wild-type amplification.

To assess the presence or absence of a particular AMR determinant, particularly when testing clinical specimens with an unknown gonococcal DNA concentration, we established the *opa* reaction for species ID as the reference reaction to determine the presence of an AMR mutation of interest. We expected that, in the presence of the target mutation, the *C_T_* values of the reference reaction (*opa*) and of the AMR determinant reaction would be similar, whereas the *C_T_* in the presence of a wild-type sequence would be substantially delayed. When testing the panel of 45 fully characterized gonococcal isolates, the *C_T_* values of the AMR reactions in the presence of the target determinants were close to the *C_T_* values of the *opa* reference reaction (Table 2). Conversely, in the presence of a wild-type sequence, we observed a delay of at least 8 additional *C_T_*s in all four AMR reactions (see Table S2 and examples in Fig. S1 in the supplemental material).

**TABLE 2:** Establishment of the Δ*C_T_*^*a*^ for each AMR reaction based on the results for the Neisseria gonorrhoeae control isolates

Target	No. of wild-type strains	Δ*C_T_* range for wild-type strains^*b*^	No. of mutant strains	Δ*C_T_* range for mutant strains	Established Δ*C_T_* for positivity
*penA* Gly545Ser	37	15.65 to >25.8	8	0.21 to 3.17	4
*penA* Asp345del	35^*c*^	9.65 to >25.25	10	−0.86 to 2.64	4
23S rRNA C2611T	43	7.60 to 12.84	2	−0.12 to −1.82	1
23S rRNA A2059G	43	9.35 to 15.20	2	0.73 to 0.76	2
*gyrA* Ser91Phe	15	13.92 to 16.93	30	1.01 to 5.78	7
16S rRNA C1192T	43	17.07 to 24.12	2	6.67 to 9.35	10

a Δ*C_T_*, difference between the *C_T_* of the AMR reaction and the *C_T_* of the *opa* reaction.

b When the *C_T_* was >40, the upper range could not be estimated precisely.

c Results for strains WHO X and WHO Z, carrying a rare mosaic *penA* allele, were considered negative and included in the range for wild-type isolates.

At the time of primer design, mosaic *penA* patterns X and XXXIV were the most frequently identified mosaic alleles. Therefore, we designed a reaction (*penA* Asp345del) targeting both these alleles, i.e., the *penA* mosaic region, including an Asp345 deletion in PBP2 compared to the sequence of nonmosaic *penA* alleles (31), and a reaction (*penA* Gly545Ser) specifically targeting the *penA* region of the Gly545Ser substitution of the *penA* mosaic XXXIV allele (32) in order to discriminate mosaic *penA* XXXIV from mosaic *penA* X alleles.

Consistently, when testing the *penA* Asp345del reaction, amplification in the presence of a mosaic *penA* X or XXXIV allele occurred with a Δ*C_T_* value of ≤2.6 compared to the reference *opa* reaction, while for nonmosaic *penA* alleles, we observed a Δ*C_T_* of ≥16.9. For the *penA* Gly545Ser reaction, amplification in the presence of the mosaic *penA* XXXIV allele occurred with a Δ*C_T_* of ≤3.2 compared to the *opa* reaction; in contrast, for the mosaic *penA* X allele, a significant delay in amplification was observed (Δ*C_T_* ≥ 19.3). However, both reactions were considered negative for mosaic *penA*-harboring strains WHO X and WHO Z (*C_T_*, >40 for the *penA* Gly545Ser reaction; Δ*C_T_*, ≥9.7 for the *penA* Asp345del reaction; Table S2), indicating that such rare H041-like mosaic *penA* alleles cannot be identified. Nevertheless, isolates harboring these alleles have remained rare (33–35). A posterior *in silico* analysis of the 22 mosaic *penA* alleles available in the NG-STAR database (36) in February 2018 also revealed that mosaic *penA* patterns XXXV, 37, 38, 59, 60, 62, 63, and 74 might not be detected with the *penA* Asp345del reaction (data not shown). For the *penA* Gly545Ser reaction, we would expect that, in addition to a mosaic *penA* XXXIV pattern, mosaic *penA* patterns 42, 51, 52, 55, and 67 would also yield a positive result, owing to the 100% sequence homology in the primer sequences.

Using the Youden index, we determined the maximum Δ*C_T_* within which the AMR reaction was considered positive (Table 2); samples with a higher Δ*C_T_* were considered not to possess the AMR determinant (examples are shown in Fig. S2). The analytical LOD for all reactions was 10 to 100 gDNA copies/reaction in the presence of the mutation of interest, whereas wild-type amplification within 40 cycles was observed only when testing ≥10^5^ gDNA copies (23S rRNA C2611T and A2059G, *gyrA* S91F) and ≥10^7^ gDNA copies (16S rRNA 1192T).

Using these cutoffs, our method showed 100% sensitivity and specificity for the prediction of phenotypic resistance to AZM and CIP in the 45 gonococcal control strains in less than 1 h (and in less than 2 h when the DNA extraction time is included) (Table S3). It should be noted that our method detects only the presence of the AZM-associated resistance mutations and not the number of mutated alleles, so we might detect AZM-susceptible gonococci with only 1 or 2 mutated alleles. However, since these strains are at risk of quickly mutating more alleles, especially if exposed to AZM selective pressure (18), it is still relevant to detect such strains and treat them as potentially AZM resistant.

Prediction of ESC resistance was less accurate (sensitivity, 62%; specificity, 86%) for three main reasons: (i) the H041-like *penA* alleles harbored in strains WHO X and WHO Z were not detected, (ii) the ESC-resistant strain WHO L possessed a nonmosaic *penA* allele, and (iii) the presence of a mosaic *penA* allele is often associated with raised MICs for ESCs which are still in the susceptible range on the basis of EUCAST criteria (21, 37). Our assay also did not predict SPC resistance in strain WHO A, which has a resistance mutation in *rpsE*. This single amino acid alteration confers low-level SPC resistance and has been only rarely described (38). The assay successfully detected the more common 16S rRNA C1192T mutation, which is associated with high-level SPC resistance, in strains WHO O and GC3.

### Cross-reaction with nongonococcal Neisseria spp.

Cross-reactivity with the N. meningitidis and commensal Neisseria spp. naturally found in the pharyngeal flora poses a great challenge in the development of molecular diagnostic tests for detecting AMR determinants in N. gonorrhoeae (20, 39). We expected that, even in the presence of 100% homology with the wild-type allele in a gonococcal species, the MAMA method would strongly inhibit background amplification from commensal sequences.

The MAMA strategy was also applied to the lower primer of the 16S rRNA reaction to improve discrimination from nongonococcal species, as described previously (39). Little or no cross-amplification was observed for the *penA* Gly545Ser and 16S rRNA C1192T reactions, when 10^7^ gDNA copies/reaction of 20 strains from different cultured Neisseria spp. were tested (Table S4). Nevertheless, cross-reactivity at a high *C_T_* (mostly comparable to those for N. gonorrhoeae wild-type sequences) was observed for the *penA* Asp345del reaction and both 23S rRNA reactions. This was due to the high similarity of the target sequences among different Neisseria spp.; notably, mosaic *penA* alleles most likely arose through recombination with the commensal orthologs (40, 41).

### Testing of N. gonorrhoeae-positive and N. gonorrhoeae-negative clinical specimens.

During our study, we collected a total of 891 ESwab samples from 261 patients, with 159 paired culture isolates being available from 128 patients. Of the 891 ESwab samples, 489 (54.9%), including 94 with paired culture isolates, met the requirements for further testing with our method (Tables S1 and S5). The 489 samples included urethral (*n* = 177), pharyngeal (*n* = 158), rectal (*n* = 130), vaginal (*n* = 13), and cervical (*n* = 11) specimens (Table S1), of which 160 were N. gonorrhoeae positive (pharyngeal, *n* = 59; rectal, *n* = 48; urethral, *n* = 46; cervical, *n* = 4; vaginal, *n* = 3) and 329 were N. gonorrhoeae negative (urethral, *n* = 131; rectal, *n* = 82; pharyngeal, *n* = 99; cervical, *n* = 7; vaginal, *n* = 10) by previous commercial NAAT testing.

When testing the NAAT-negative samples, nonspecific amplification of the *opa* reaction was observed in most samples of all specimen types (i.e., the proportions of samples with an *opa C_T_* of >40 were 11.1%, 18.3%, 18.3%, 25%, and 0% for pharyngeal, urethral, rectal, vaginal, and cervical specimens, respectively), possibly due to sample degradation, suboptimal primer specificities/PCR conditions, and/or background amplification from commensals. The potential of background amplification for *opa* was also confirmed when testing the panel of nongonococcal control strains at 10^7^ gDNA copies/reaction (i.e., cross-reactivity was observed for N. meningitidis, N. mucosa, and N. weaveri; Table S4). For these reasons, we analyzed the distributions of the *opa C_T_* values for specimens with positive and negative commercial NAAT results in order to determine the optimal cutoff point (i.e., the maximum *C_T_* value for which the sample would be considered positive for the presence of N. gonorrhoeae). We initially evaluated the addition of the *porA* reaction as a confirmatory identification reaction. However, we eventually dismissed it since this reaction did not perform well and led to inconsistent results with the *opa* reaction (data not shown).

Overall, using the optimal cutoffs for *opa* (e.g., a *C_T_* of <26.1 for urethral samples; Table 3), our method showed good agreement with the reference NAATs for the detection of N. gonorrhoeae in genital specimens (e.g., a sensitivity of 93% and a specificity of 100% for urethral samples), but additional testing on different sample sets will be required in the future to confirm the accuracy of the established cutoffs. The real-time PCR was less accurate in identifying N. gonorrhoeae in extragenital specimens. In particular, the low specificity resulting in a low positive predictive value indicates that further optimization of the method is needed to increase the accuracy for these sample types (Table 3).

**TABLE 3:** Optimal *opa C_T_* cutoff value and performance of the real-time PCR for Neisseria gonorrhoeae identification compared to that of commercial NAATs^*a*^

Sample site	*opa* cutoff	No. of isolates with the following result:					Sensitivity (95% CI)^*b*^	Specificity (95% CI)^*b*^
		TP	FP	FN	TN	Total		
Urethra	26.1	43	0	3	131	177	0.93 (0.82, 0.99)	1.00 (0.97, 1.00)
Rectum	33.7	46	12	2	70	130	0.96 (0.86, 0.99)	0.85 (0.76, 0.92)
Pharynx	31.3	53	9	6	90	158	0.90 (0.79, 0.96)	0.91 (0.83, 0.96)
Cervix	28.4	4	0	0	7	11	1.00 (0.4, 1.00)	1.00 (0.59, 1.00)
Vagina	33.4	3	1	0	9	13	1.00 (0.29, 1.00)	0.90 (0.55, 1.00)
All sites combined	33	149	22	11	307	489	0.92 (0.87, 0.96)	0.91 (0.87, 0.94)

a CI, confidence interval; TP, true positive; FP, false positive; FN, false negative; TN, true negative.

b Sensitivity and specificity were calculated using the results of the commercial NAATs as the reference standard.

All 94 samples from 77 patients for which paired culture isolates were available tested positive for N. gonorrhoeae by using our real-time PCR (Tables S1 and S5). Additionally, our method accurately predicted (directly from the clinical ESwab samples) the susceptibility profiles for CIP, AZM, SPC, and ESCs of the paired culture isolates, as determined by comparison with the MICs as the gold standard (Table 4).

**TABLE 4:** Performance of the real-time PCR in predicting antimicrobial resistance to four antibiotic classes directly from Neisseria gonorrhoeae-positive clinical specimens with paired N. gonorrhoeae isolates^*a*^

Reaction	Associated antibiotic	No. of isolates with the following result:					Sensitivity (95% CI)^*b*^	Specificity (95% CI)^*b*^
		TP	FP	FN	TN	Total		
*penA* Asp345del ± *penA* Gly545Ser	ESCs	1^*c*^	17^*d*^	0	76	94	1.00 (0.03, 1.00)	0.82 (0.72, 0.89)
23S rRNA C2611T and/or 23S rRNA A2059G	AZM	1	1	0	92	94	1.00 (0.03, 1.00)	0.99 (0.94, 1.00)
*gyrA* Ser91Phe	CIP	41	1	0	52	94	1.00 (0.91, 1.00)	0.98 (0.90, 1.00)
16S rRNA C1192T	SPC	0	2	0	92	94	NA	0.98 (0.93, 1.00)

a CI, confidence interval; TP, true positive; FP, false positive; FN, false negative; TN, true negative; ESCs, extended-spectrum cephalosporins; AZM, azithromycin; CIP, ciprofloxacin; SPC, spectinomycin; NA, not applicable.

b Sensitivity and specificity were calculated on the basis of the susceptibility phenotype (MICs) of the paired culture isolates.

c This isolate harbored a *penA* mosaic X allele and was correctly identified as ESC resistant by the *penA* Ala345del reaction.

d Three isolates were correctly predicted to harbor a mosaic *penA* allele on the basis of the molecular characterization but were susceptible to ESCs.

Our assay predicted CIP resistance with a 100% sensitivity and a 98% specificity. One clinical sample that tested positive by the mosaic *penA* reaction (*penA* Asp345del) had a paired isolate that carried a mosaic *penA* X allele and was phenotypically resistant to CFM (28). This isolate was subjected to PCR/sequencing (23), confirming the presence of a mosaic *penA* X allele. Similarly, all three samples that tested positive for both *penA* Asp345del and *penA* Gly545Ser had paired isolates that contained the mosaic *penA* XXXIV allele, despite being phenotypically susceptible to ESCs according to the EUCAST criteria. This is not surprising, since strains harboring this particular allele often show only raised ESC MICs which are still in the susceptible range (37). Nevertheless, the detection of such strains is still of clinical relevance, since isolates harboring this mosaic *penA* variant (e.g., the internationally spreading clone NG-MAST ST1407) have been associated with CFM treatment failures, despite being susceptible on the basis of standard phenotypic testing (42). Overall, our assay showed a 100% sensitivity and a 98% to 100% specificity for the prediction of the AMR phenotype for all reactions except the *penA* Asp345del reaction (82% specificity). False-positive results were mainly associated with the *penA* Asp345del reaction in extragenital specimens (i.e., likely due to the background amplification of other Neisseria spp.). In fact, the specificities of the *penA* Asp345del reaction observed for genital and extragenital specimens were 95% and 72%, respectively (Tables 5 and 6).

**TABLE 5:** Performance of the real-time PCR in predicting antimicrobial resistance to four antibiotic classes directly from Neisseria gonorrhoeae-positive genital specimens with paired N. gonorrhoeae isolates^*a*^

Reaction	Associated antibiotic	No. of isolates with the following result:					Sensitivity (95% CI)^*b*^	Specificity (95% CI)^*b*^
		TP	FP	FN	TN	Total		
*penA* Asp345del ± *penA* Gly545Ser	ESCs	1^*c*^	2^*d*^	0	37	40	1.00 (0.03, 1.00)	0.95 (0.83, 0.99)
23S rRNA C2611T and/or 23S rRNA A2059G	AZM	1	0	0	39	40	1.00 (0.03, 1.00)	1.00 (0.91, 1.00)
*gyrA* Ser91Phe	CIP	18	0	0	22	40	1.00 (0.81, 1.00)	1.00 (0.85, 1.00)
16S rRNA C1192T	SPC	0	0	0	40	40	NA	1.00 (0.91, 1.00)

a CI, confidence interval; TP, true positive; FP, false positive; FN, false negative; TN, true negative; ESCs, extended-spectrum cephalosporins; AZM, azithromycin; CIP, ciprofloxacin; SPC, spectinomycin; NA, not applicable.

b Sensitivity and specificity were calculated on the basis of the susceptibility phenotype (MICs) of the paired culture isolates.

c This isolate harbored a *penA* mosaic X allele and was correctly identified as ESC resistant by the *penA* Asp345del reaction.

d One isolate was correctly predicted to harbor a mosaic *penA* allele on the basis of the molecular characterization but was susceptible to ESCs.

**TABLE 6:** Performance of the real-time PCR in predicting antimicrobial resistance to four antibiotic classes directly from Neisseria gonorrhoeae-positive extragenital specimens with paired N. gonorrhoeae isolates^*a*^

Reaction	Associated antibiotic	No. of isolates with the following result:					Sensitivity (95% CI)^*b*^	Specificity (95% CI)^*b*^
		TP	FP	FN	TN	Total		
*penA* Asp345del ± *penA* Gly545Ser	ESCs	0	15^*c*^	0	39	54	NA	0.72 (0.58, 0.84)
23S rRNA C2611T and/or 23S rRNA A2059G	AZM	0	1	0	53	54	NA	0.98 (0.90, 1.00)
*gyrA* Ser91Phe	CIP	23	1	0	30	54	1.00 (0.85, 1.00)	0.97 (0.83, 1.00)
16S rRNA C1192T	SPC	0	2	0	52	54	NA	0.96 (0.87, 1.00)

a CI, confidence interval; TP, true positive; FP, false positive; FN, false negative; TN, true negative; ESCs, extended-spectrum cephalosporins; AZM, azithromycin; CIP, ciprofloxacin; SPC, spectinomycin; NA, not applicable.

b Sensitivity and specificity were calculated on the basis of the susceptibility phenotype (MICs) of the paired culture isolates.

c Two isolates were correctly predicted to harbor a mosaic *penA* allele on the basis of the molecular characterization but were susceptible to ESCs.

A main limitation of the present study was that we did not identify clinical samples positive for some AMR mutations (i.e., 23S rRNA A2059G and 16S rRNA C1192T), since they are still very rare (15), and only a few positive results for additional AMR determinants (i.e., 23S rRNA C2611T and mosaic *penA* alleles) were recorded. In this context, the results obtained from the remaining 55 samples, for which no culture isolates were available, also suggest that the number of samples testing positive for ESC, AZM, and SPC resistance overestimates the presence of these resistance determinants in extragenital specimens (Table S6). This finding likely results from false-positive results due to cross-reactivity with other Neisseria spp. (Tables S4). Additionally, lower gonococcal loads in extragenital specimens, also typically associated with the inability to recover culture isolates, may also lead to a higher *opa C_T_* and, thus, a decreased Δ*C_T_* (Fig. S3; Table S7). Finally, samples were processed at different times after collection and could not be tested fresh, potentially allowing degradation.

Overall, further evaluations will be beneficial to confirm the performance of our method with genital specimens, and in particular, more sample sets with different AMR determinants should be included. Our results also show that the method does not accurately predict AMR in extragenital samples.

We designed a SYBR green approach since it is inexpensive, it requires only specific primers for the different reactions, and it is more feasible for application in low-income settings; in addition, it is advantageous for translation into a point-of-care test. However, in light of our results, the implementation of molecular probes to increase the specificity may represent a useful future optimization strategy.

### Conclusions.

We developed a rapid multitarget SYBR green-based method that was successfully applied to accurately identify and predict the N. gonorrhoeae antimicrobial resistance phenotype directly from urogenital samples in transport medium, which can be also used to perform culture isolation concomitantly. However, our data showed that the method cannot be used for some extragenital specimens, particularly pharyngeal samples, because of the lower gonococcal load, the higher risk of DNA degradation, and the cross-reactivity with other Neisseria spp. Nevertheless, owing to its ease of use and rapidity, this method might be a promising candidate for further development into a rapid test. Such a test, if deployed at the point of care, could contribute to individually tailored gonorrhea treatments in the future.

## Supplementary Material

Supplemental file 1

Supplemental file 2
