# Supplementary material for: Mismatch Amplification Mutation Assay-Based Real-Time PCR for Rapid Detection of Neisseria gonorrhoeae and Antimicrobial Resistance Determinants in Clinical Specimens
Source: J Clin Microbiol. 2018 Aug 27;56(9):e00365-18. doi: 10.1128/JCM.00365-18 (PMC6113480; doi:10.1128/JCM.00365-18)
Supplement: Supplemental file 1 [file zjm999096088s1.pdf]

Table S1. Samples included in the study

**Samples**	**urethra**	**rectum**	**pharynx**	**cervix**	**vagina**	**total**
NAAT result available	259	259	261	19	21	819
RT-PCR results available	177	130	158	11	13	489
Paired culture isolates	57	58	35	5	4	159
RT-PCR and paired culture isolates available	35	31	23	3	2	94

Table S2. Real-time PCR results for the 45 *Neisseria gonorrhoeae* (NG) control isolates

**NG control isolates**	*opa* Ct±SD [a]	*penA* Asp345del Ct±SD [a]	*penA* Gly545Ser Ct±SD [a]	23S rRNA A2059G Ct±SD [a]	23S rRNA C2611T Ct±SD [a]	*gyrA* Ser91Phe Ct±SD [a]	16S rRNA C1192T Ct±SD [a]
2131245	15.14±0.06	36.81±NA	38.83±NA	28.21±0.08	25.75±0.03	18.69±0.13	35.97±0.53
2132307	19.71±1.67	18.91±0.81	20.01±0.73	29.06±0.97	27.31±1.24	21.5±0.17	39.01±0.88
2112655	19.09±0.56	19.76±0.13	20.85±0.38	31.59±0.38	29.38±0.11	22.24±0.19	38.76±0.33
2086772	18.74±0.06	33.83±0.49	34.39±0.5	31.35±0.03	29.03±0.06	21.98±0	38.29±0.32
1946253	19.66±0.11	19.45±0.11	20.26±0.01	30.69±0.23	28.23±0.02	21.11±0.05	37.55±0.01
1954615	17.19±0.03	34.2±0.44	35.76±0.68	28.88±0.03	26.69±0.02	19.53±0.14	37.36±0.69
2129562	20.69±0.01	>40±NA	>40±NA	32.18±0.09	29.48±0.05	22.36±0.15	39.791±NA
2121127	20.48±0.01	>40±NA	>40±NA	33.42±0.12	30.89±0.01	24.35±0.07	>40±NA
1944251	18.35±0.07	38.21±NA	>40±NA	31.28±0.03	29.01±0.05	33.53±0.13	38.81±0.11
2036196	18.7±0.08	>40±NA	>40±NA	31.74±0.01	29.31±0.11	22.29±0.04	38.58±0.17
2119347	19.12±0.59	18.59±0.23	19.44±0.06	29.48±0.04	26.85±0.03	20.13±0.13	36.19±0.03
2096513	15.32±0.11	36.199±1.05	37±NA	28.73±0.11	25.92±0.05	30.25±0.03	35.41±0.1
1929711	16.28±0.15	>40±NA	37.68±0.69	29.37±0.07	26.6±0.03	30.83±0.05	35.73±0.38
1865296	16.99±0.05	37.17±NA	>40±NA	27.72±0.01	25.16±0.02	18.04±0.03	34.25±0.44
1820606	15.7±0.18	>40±NA	36.37±NA	28.47±0.1	25.94±0.08	30.33±0.12	35.16±0.02
1922603	18.28±0.1	18.57±0.05	19.6±0.1	29.88±0.13	27.66±0.05	20.57±0.06	38.55±0.58
1912168	14.82±0.06	31.71±0.28	32.77±0.26	27.71±0.13	25.29±0.01	18.07±0.01	36.51±0.34
2017570	12.11±2.25	14.13±0.14	15.28±0.18	25.06±1.43	23.44±0.56	15.37±1.04	33.17±1.28
1996003	17.46±0.05	>40±NA	>40±NA	29.48±0.01	27.38±0.02	20.11±0.07	37.1±0.15
2004277	17.93±0.89	>40±NA	>40±NA	30.58±0.84	28.21±0.81	33.65±0.17	39.28±0.34
1973517	16.2±0.04	35.95±NA	39.3±0.58	30.72±0.13	28.14±0.33	33.13±0.04	39.5±NA
1832086	18.92±0.01	18.06±0.3	19.16±0.1	29.61±0.01	27.34±0.01	20.2±0.09	37.15±0.26
1872778	16.43±0.06	37.26±1.3	37.27±0.81	28.46±0.09	25.94±0.24	18.69±0.3	35.8±0.03

1910312	14.92±0.03	36.56±0.46	37.66±NA	27.89±0.07	25.55±0.04	29.99±0.1	35.03±0.18
1859198	17.31±0.07	33.72±0.67	35.33±1.43	28.93±0.04	26.69±0.02	19.57±0.06	35.77±0.36
1869994	16.84±0.03	35.52±0.04	36.14±0.48	29.71±0.08	27.37±0.03	20.46±0.06	36.73±0.11
WHO A	16.8±0.27	>40±NA	37.92±NA	30.02±0.03	27.72±0.06	32.38±0.03	39.65±NA
WHO O	17.1±0.27	37±NA	>40±NA	31.28±0.05	29±0.05	33.7±0.28	26.448±0.18
WHO L	16.15±0.02	37.07±NA	37.66±NA	30.37±0.11	27.97±0	20.52±0.03	39.48±0.68
WHO K	14.16±2.16	16.8±1.22	36.25±0.26	29.36±0.15	27±0.22	19.94±0.33	38.28±0.24
G07	14.86±0.13	35.11±0.37	36.8±NA	26.46±0.01	13.04±0.12	16.77±0.13	34.02±0.51
WHO V	17.37±0.1	36.85±NA	38.67±NA	18.1±0.02	27.48±0.02	20.49±0.02	37.38±0.01
WHO Y	17.97±0.07	17.61±0.12	18.18±0.07	28.77±0.07	26.31±0.01	19.17±0.09	35.47±0.05
ATCC49226	16.3±0.02	36.98±NA	37.16±NA	28.62±0.01	26.25±0.02	30.22±0.04	35.49±0.28
WHO P	14.37±0.13	37.22±0.11	37.49±1.83	27.33±0.12	24.86±0.01	29.15±0.06	34.14±0.06
WHO Z	13.97±0.01	35.77±0.02	>40±NA	26.48±0.02	23.62±0.02	17.05±0.08	33.61±0.4
WHO U	13.41±2.63	37.08±NA	38.09±NA	26.83±0.6	13.29±1.28	29.03±0.34	33.87±0.4
GC3	13.52±0.84	37.85±NA	>40±NA	26.72±0.73	24.28±0.47	28.91±0.21	20.18±0.09
GC4	12.33±0.08	37.58±0.84	38.13±NA	13.09±0.04	22.5±0.01	26.99±0.05	31.67±0.31
WHO F	11.8±0.01	>40±NA	>40±NA	24.84±0.04	22.63±0.17	27.2±0.03	31.81±0.52
WHO G	12.24±0.05	>40±NA	>40±NA	25.3±0.1	22.46±0.01	15.64±0.12	31.7±0.34
WHO M	11.33±0.13	>40±NA	>40±NA	24.34±0.22	23.25±0.23	16.11±0.1	31.98±0.52
WHO N	12.57±0.03	>40±NA	>40±NA	25.18±0.12	22.32±0.05	15.68±0.1	31.24±0.08
WHO W	12.13±0.11	13.87±0.03	31.47±0.01	25.34±0.06	22.6±0.08	15.75±0.14	31.09±035
WHO X	11.96±2.9	21.61±0.78	>40±NA	25.26±1.89	22.21±0.41	15.95±0.34	31.17±0.27

Note. Ct, cycle threshold; SD, standard deviation; NA, not applicable

[a] Average cycle threshold and standard deviation of duplicates are shown; if only one duplicate had a Ct below 40, the Ct of the duplicate with Ct<40 is shown

Table S3. Performance of the real-time PCR in the prediction of antimicrobial resistance in *Neisseria gonorrhoeae* (NG) control isolates

**Reaction**	**Associated antibiotic**	**TP**	**FP**	**FN**	**TN**	**Total**	**Sensitivity (95% CI)** [a]	**Specificity (95% CI)** [a]
penA Asp345del +/- *penA* Gly545Ser	ESCs	5	5	3	32	45	0.62 (0.24, 0.91)	0.86 (0.71, 0.95)
23S rRNA C2611T and/or 23S rRNA A2059G	AZM	4	0	0	41	45	1.00 (0.40, 1.00)	1.00 (0.91, 1.00)
gyrA Ser91Phe	CIP	30	0	0	15	45	1.00 (0.88, 1.00)	1.00 (0.78, 1.00)
16S rRNA C1192T	SPC	2	0	1	42	45	0.67 (0.09, 0.99)	1.00 (0.92, 1.00)

Note. CI, confidence interval; TP, true positive; FP, false positive; FN, false negative; TN, true negative; ESCs, extended-spectrum cephalosporins; AZM, azithromycin; CIP, ciprofloxacin; SPC, spectinomycin

[a] Sensitivity and specificity were calculated based on the phenotype (MICs) of the NG control isolates

Table S4. Real-time PCR results for the non-gonococcal control isolates

**Non-gonoccal isolates**	*opa* Ct±SD [a]	*penA* Asp345del Ct±SD [a]	*penA* Gly545Ser Ct±SD [a]	23S rRNA A2059G Ct±SD [a]	23S rRNA C2611T Ct±SD [a]	*gyrA* Ser91Phe Ct±SD [a]	16S rRNA C1192T Ct±SD [a]
N. sicca (3319)	>40±NA	30.62±0.03	>40±NA	33.19±0.01	31.08±0.21	>40±NA	>40±NA
N. mucosa (130/93)	31.75±0.09	31.46±0.02	>40±NA	30.49±0.1	28.29±0.06	>40±NA	>40±NA
N. sicca (3754)	37.41±0.88	31.03±0.26	>40±NA	31.79±1.07	29.81±0.71	>40±NA	>40±NA
N. sicca (PS-7)	35.02±0.33	28.52±0	36.732±NA	26.94±0.24	23.91±0.16	>40±NA	>40±NA
N. flavescens (4754)	>40±NA	>40±NA	>40±NA	35.32±0.41	30.55±0.01	>40±NA	>40±NA
N. flava (10d)	>40±NA	19.05±0.12	>40±NA	34.65±0.21	>40±NA	>40±NA	>40±NA
N. subflava (112-94)	>40±NA	19.9±0.07	>40±NA	34.75±0.32	29.21±0.04	>40±NA	>40±NA
N. flavescens (423)	>40±NA	18.81±0	>40±NA	29.98±0.19	27.71±0.01	>40±NA	>40±NA
N. subflava (82-94)	>40±NA	21.29±0.26	35.82±NA	36.46±0.04	30.62±0.15	>40±NA	>40±NA
N. polysaccharea (N462)	33.5±1.64	31.58±1.22	>40±NA	38.55±0.5	32.95±0.15	>40±NA	>40±NA
N. flavescens (107)	>40±NA	33.36±0.82	>40±NA	35.13±0.9	32.3±0.01	>40±NA	>40±NA
N. lactamica (RS-46)	>40±NA	32.74±0.39	>40±NA	29.29±0.44	34.79±0.41	37.2±NA	>40±NA
N. cinerea (58/95)	>40±NA	37.86±0.26	>40±NA	39.38±NA	33±0.1	>40±NA	>40±NA
N. cinerea (145/96)	>40±NA	>40±NA	>40±NA	34.62±0.01	32.76±0.07	>40±NA	>40±NA
N. mucosa (179/98)	>40±NA	>40±NA	37.4±NA	33±0.55	29.69±1.46	>40±NA	>40±NA
N. meningitidis (5408.62)	37.6±1.25	>40±NA	>40±NA	31.36±0.07	34.11±0.2	31.27±0.12	>40±NA
N. meningitidis (5507.19)	34.12±0.13	>40±NA	>40±NA	29.53±0.18	23.94±0.01	29.9±0.04	>40±NA
N. meningitidis (4904.61)	34.16±0.8	38.36±NA	>40±NA	29.52±0	23.96±0.12	29.73±0.04	>40±NA
N. weaveri (3803.40)	30.32±2.16	34.27±0.06	>40±NA	25.74±0.1	36.4±0.05	35.2±0.42	>40±NA
N. bacilliformis (A16.28)	34.91±0.09	39.36±0.25	>40±NA	26.77±0.03	34.52±1.45	36.91±0.11	32.72±0.05

Note. Ct, cycle threshold; SD, standard deviation; NA, not applicable

[a] Average cycle threshold and standard deviation of duplicates are shown; if only one duplicate had a Ct below 40, the Ct of the duplicate with Ct<40 is shown

Table S5. MIC results for the paired culture isolates

Isolate number	MIC (mg/L)				
	Ceftriaxone	Cefixime	Ciprofloxacin	Spectinomycin	Azithromycin
1	<0.002	<0.016	0.003	12	0.38
2	0.016	0.023	6	12	0.38
3	0.003	<0.016	0.003	12	0.125
4	<0.002	<0.016	0.003	8	0.38
5	<0.002	<0.016	0.002	8	0.25
6	<0.002	<0.016	0.002	8	0.094
7	0.002	<0.016	1.5	12	0.19
8	0.003	<0.016	1.5	12	0.19
9	0.003	<0.016	0.003	12	0.19
10	<0.002	<0.016	0.003	8	0.38
11	0.003	<0.016	0.003	6	0.38
12	0.016	0.032	16	6	0.25
13	<0.002	<0.016	0.002	6	0.19
14	0.023	<0.016	>32	4	0.25
15	0.023	<0.016	0.006	16	0.75
16	<0.002	<0.016	0.002	4	0.064
17	0.023	<0.016	16	8	0.19
18	<0.002	<0.016	3	8	0.19
19	0.002	<0.016	0.002	8	0.064
20	0.002	<0.016	3	12	0.125
21	0.003	<0.016	2	12	0.19
22	0.004	<0.016	2	12	0.25
23	0.016	0.047	>32	6	0.5
24	0.003	<0.016	0.003	8	0.094
25	0.003	<0.016	0.003	12	0.19
26	0.003	<0.016	0.003	8	0.19
27	0.006	<0.016	0.002	8	0.19
28	0.004	<0.016	0.002	8	0.19
29	<0.002	<0.016	0.003	8	0.094
30	<0.002	<0.016	0.003	12	0.125
31	0.002	<0.016	0.094	8	0.38
32	<0.002	<0.016	0.003	6	0.19
33	<0.002	<0.016	0.003	8	0.19
34	<0.002	<0.016	0.003	8	0.19
35	0.032	0.047	6	8	0.19
36	<0.002	<0.016	0.003	12	0.19
37	0.004	<0.016	0.008	12	0.19
38	0.047	0.047	12	16	0.19
39	0.032	0.047	8	12	0.19
40	<0.002	<0.016	0.004	24	0.38
41	0.004	<0.016	0.003	8	0.19
42	<0.002	<0.016	0.002	6	0.19

43	0.002	<0.016	1.5	12	0.25
44	<0.002	<0.016	0.003	8	0.25
45	0.002	<0.016	0.002	8	0.25
46	0.004	<0.016	0.006	8	0.5
47	0.002	<0.016	0.003	12	0.125
48	0.008	<0.016	0.008	12	0.5
49	<0.002	<0.016	0.003	12	0.094
50	0.023	0.047	16	12	0.25
51	0.006	<0.016	0.003	12	0.19
52	0.002	<0.016	1.5	12	0.38
53	0.008	<0.016	0.008	12	0.75
54	0.016	0.016	6	12	0.19
55	0.006	<0.016	0.003	12	0.19
56	0.006	<0.016	0.003	16	0.25
57	0.006	<0.016	>32	12	1.5
58	0.002	<0.016	0.003	8	0.19
59	0.016	0.032	12	12	0.38
60	0.002	<0.016	<0.002	8	0.047
61	0.006	<0.016	2	16	0.25
62	0.003	<0.016	0.003	12	4
63	0.004	<0.016	0.004	12	0.38
64	0.003	<0.016	4	12	0.19
65	0.003	<0.016	0.004	8	0.125
66	0.004	<0.016	6	12	1
67	0.003	<0.016	0.004	8	0.19
68	0.004	<0.016	0.004	12	0.19
69	0.004	<0.016	0.004	16	0.38
70	0.002	<0.016	0.004	12	0.25
71	0.002	<0.016	0.004	8	0.19
72	0.016	0.064	>32	12	0.75
73	0.032	0.094	>32	16	0.75
74	0.023	0.094	>32	12	0.75
75	0.002	<0.016	3	12	0.19
76	0.004	<0.016	4	12	0.19
77	0.002	<0.016	4	8	0.125
78	0.004	<0.016	<0.002	8	0.047
79	0.032	0.047	>32	12	0.38
80	0.047	0.047	>32	12	0.38
81	0.047	0.047	>32	12	0.38
82	0.032	0.064	>32	12	0.5
83	0.047	0.047	>32	12	0.5
84	0.023	0.047	>32	12	0.5
85	0.002	<0.016	0.004	12	0.25
86	0.006	0.016	0.19	12	0.064
87	0.002	<0.016	0.003	12	0.38
88	0.003	<0.016	6	16	0.25

89	0.002	<0.016	0.004	12	0.38
90	0.023	0.032	12	12	0.38
91	0.023	0.19	>32	12	0.094
92	0.012	0.032	0.006	6	0.023
93	0.006	0.016	2	8	0.19

*There are 93 instead of 94 results because the specimens for 4 P and 4 R were pooled together for culture.

Table S6. Prediction of antimicrobial resistance based on the real-time PCR results for samples without paired culture isolates

Reaction	Associated antibiotic	Genital specimens			Extra-genital specimens			All sites combined		
		resistant	susceptible	total	resistant	susceptible	total	resistant	susceptible	total
penA Asp345del +/- *penA* Gly545Ser	ESCs	0	10	10	25	20	45	25	30	55
23S rRNA C2611T and/or 23S rRNA A2059G	AZM	0	10	10	7	38	45	7	48	55
gyrA Ser91Phe	CIP	2	8	10	18	27	45	20	35	55
16S rRNA C1192T	SPC	0	10	10	2	43	45	2	53	55

Note. ESCs, extended-spectrum cephalosporins; AZM, azithromycin; CIP, ciprofloxacin; SPC, spectinomycin

Table S7. Clinical samples per sampling site with and without paired isolates

**Sample site**	**Samples with paired isolates (95% CI)**	**Samples without paired isolates (95% CI)**
pharynx	23 (0.10, 0.36)	30 (0.40, 0.68)
rectum	31 (0.20, 0.45)	15 (0.10, 0.41)
urethra	35 (0.30, 0.49)	8 (0.00, 0.28)
vagina	2 (0.00, 0.14)	1 (0.00, 0.15)
cervix	3 (0.00, 0.15)	1 (0.00, 0.15)

Note. CI, multinomial confidence interval of the proportion
