# Supplementary material for: Mismatch Amplification Mutation Assay-Based Real-Time PCR for Rapid Detection of Neisseria gonorrhoeae and Antimicrobial Resistance Determinants in Clinical Specimens
Source: J Clin Microbiol. 2018 Aug 27;56(9):e00365-18. doi: 10.1128/JCM.00365-18 (PMC6113480; doi:10.1128/JCM.00365-18)
Supplement: Supplemental file 2 [file zjm999096088s2.pdf]

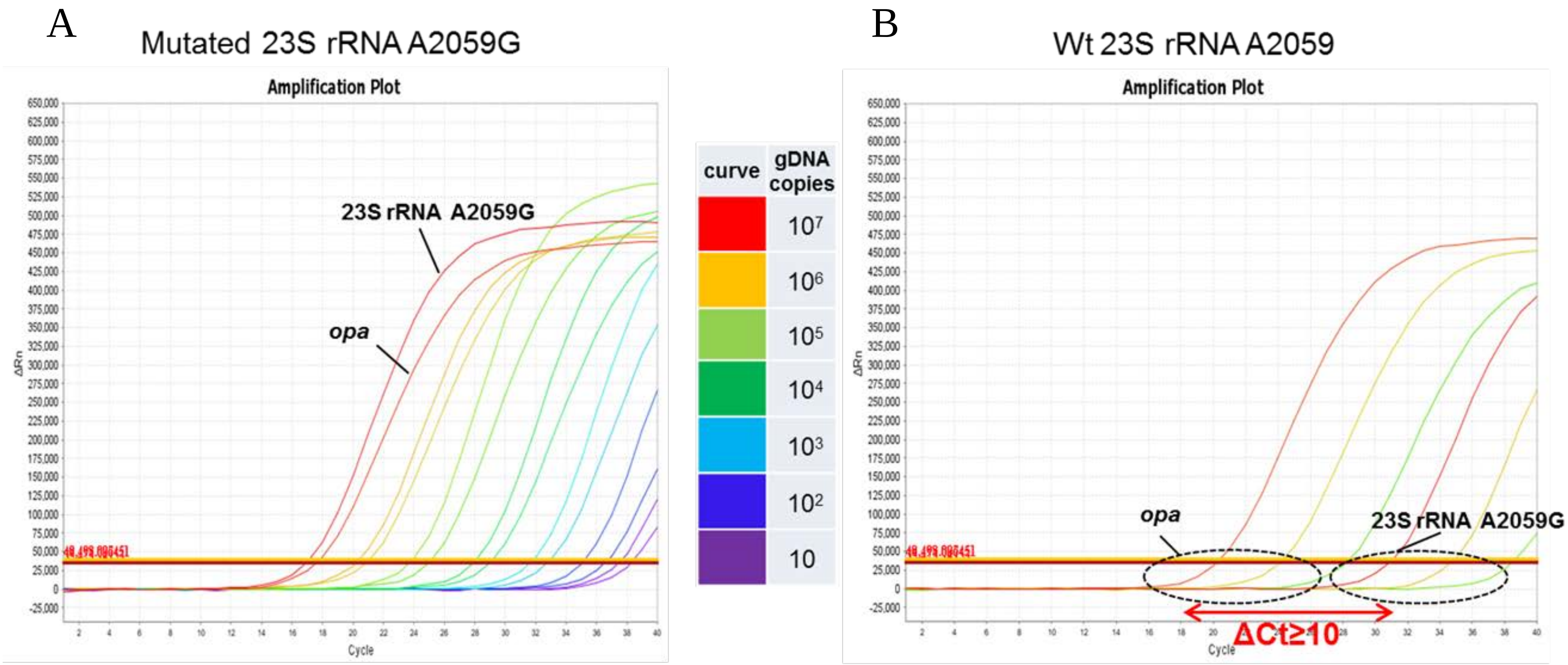

Figure S1. Example of delayed amplification due to the MAMA-strategy. When plating same amounts of gDNA copies/reaction, the Ct of the *opa* reaction will be comparable to the the Ct of the AMR reaction if the mutated allele is present (**A**), whereas there will be a strong delay in the amplification if the wild type allele is present (**B**). The different colors indicate different amounts of plated gDNA.

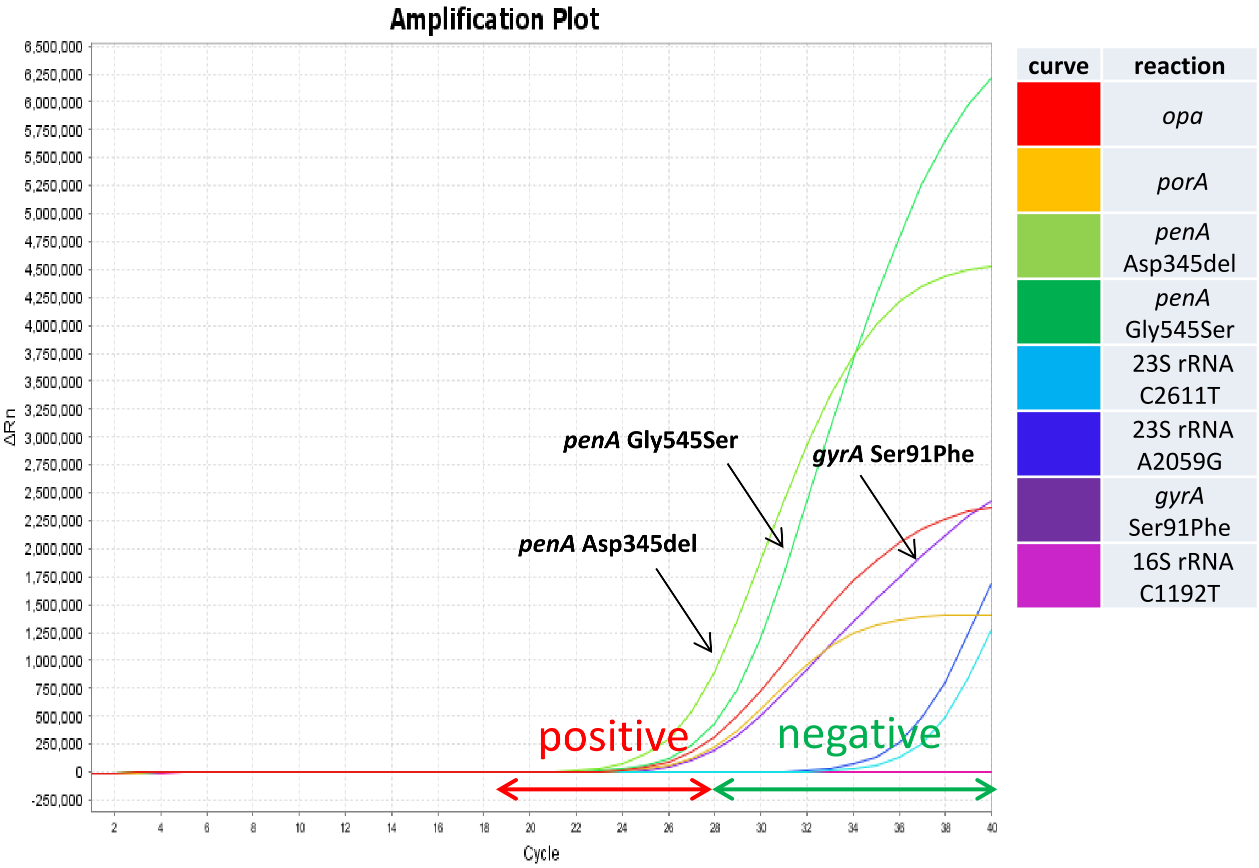

Figure S2. Example of a sample positive for NG. Different reactions are highlighted with different colors. Based on the ΔCt established for the AMR reactions, this sample was positive for the both *penA* reactions, indicating the presence of a mosaic XXXIV penA allele, as well as for the *gyrA* Ser91Phe reaction. All other AMR reactions were negative.

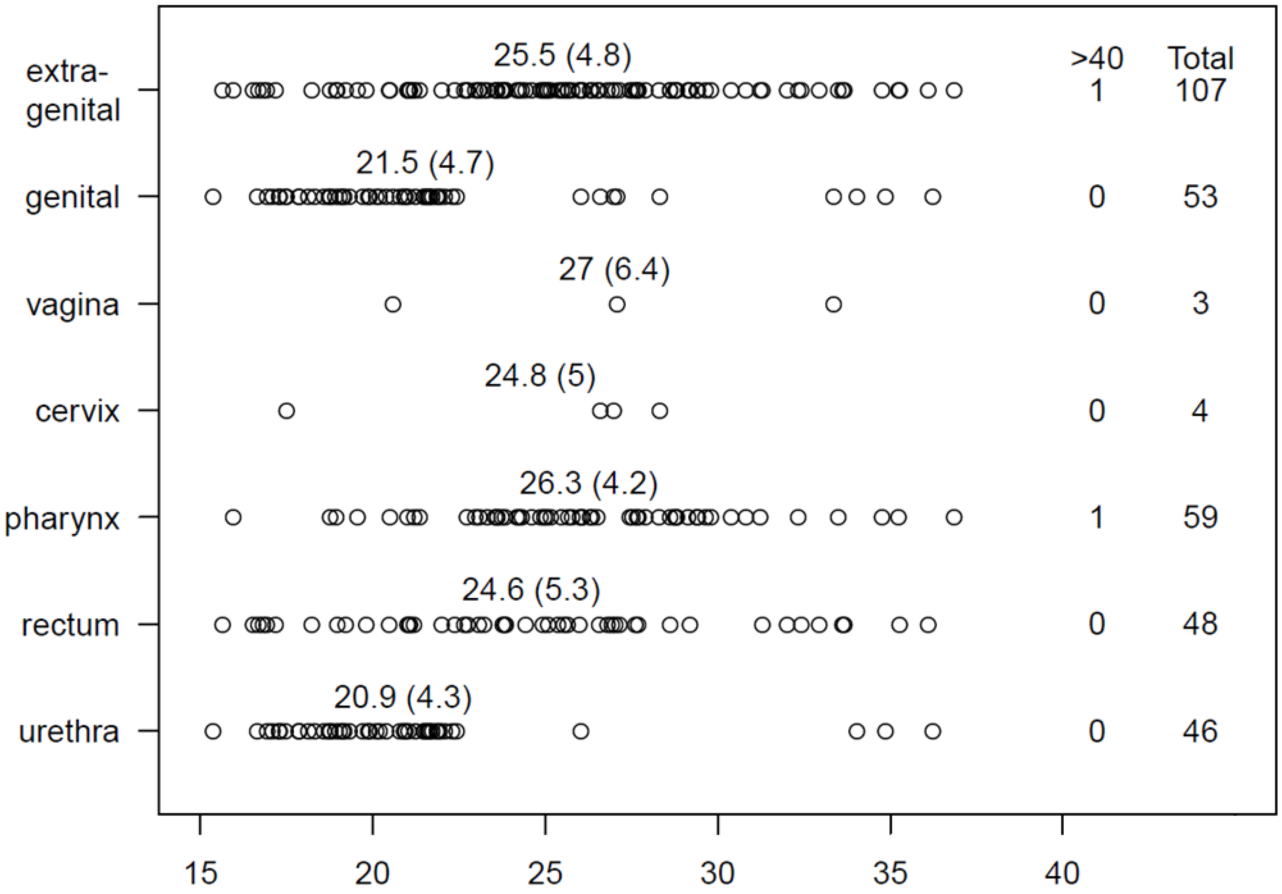

Figure S3. Distribution of the cycle threshold values of the *opa* reaction obtained for each sample type positive for NG by commercial NAAT. Each dot represents one sample. The mean value for each sample type is shown on top with the brackets denoting the standard deviation.
